# Correlation between blood glucose and cerebrospinal fluid glucose levels in patients with differences in glucose metabolism

**DOI:** 10.3389/fneur.2023.1103026

**Published:** 2023-04-27

**Authors:** Qing Che Tan, Xiao Wei Xing, Jia Tang Zhang, Mian Wang He, Yu Bao Ma, Lei Wu, Xiaolin Wang, Hong Fen Wang, Sheng Yuan Yu

**Affiliations:** ^1^Medical School of Chinese PLA, Beijing, China; ^2^First Affiliated Hospital of Chinese PLA General Hospital, Beijing, China; ^3^Hainan Branch of People’s Liberation Army General Hospital, Sanya, China

**Keywords:** correlation, cerebrospinal fluid glucose, blood glucose, central nervous system infection, CSF/blood glucose ratio, abnormal glucose metabolism, normal glucose metabolism

## Abstract

**Objectives:**

We aimed to determine a method to identify normal cerebrospinal fluid (CSF) glucose levels by examining the correlation between blood and CSF glucose levels in patients with normal and abnormal glucose metabolism.

**Methods:**

One hundred ninety-five patients were divided into two groups according to their glucose metabolism. The glucose levels were obtained from CSF and fingertip blood at 6, 5, 4, 3, 2, 1, and 0  h before lumbar puncture. SPSS 22.0 software was used for the statistical analysis.

**Results:**

In both the normal and abnormal glucose metabolism groups, CSF glucose levels increased with blood glucose levels at 6, 5, 4, 3, 2, 1, and 0  h before lumbar puncture. In the normal glucose metabolism group, the CSF/blood glucose ratio range was 0.35–0.95 at 0–6  h before lumbar puncture, and the CSF/average blood glucose ratio range was 0.43–0.74. In the abnormal glucose metabolism group, the CSF/blood glucose ratio range was 0.25–1.2 at 0–6  h before lumbar puncture, and the CSF/average blood glucose ratio range was 0.33–0.78.

**Conclusion:**

The CSF glucose level is influenced by the blood glucose level 6  h before lumbar puncture. In patients with normal glucose metabolism, direct measurement of the CSF glucose level can be used to determine whether the CSF level is normal. However, in patients with abnormal or unclear glucose metabolism, the CSF/average blood glucose ratio should be used to determine whether the CSF glucose level is normal.

## Introduction

Cerebrospinal fluid (CSF) examination is important for the diagnosis of central nervous system (CNS) diseases. A decline in the CSF glucose level is an important indicator for diagnosis of CNS infection and carcinoma. The CSF glucose level is influenced by the serum glucose level. Fluctuations in blood glucose parallel changes in the CSF glucose (1, 2). Low CSF glucose can be masked by high blood glucose, and low CSF glucose during low blood glucose maybe misinterpreted as CNS infection. This misleading effect can be corrected by estimating the CSF/blood glucose (3). Therefore, the CSF/blood glucose ratio was used to determine whether a patient has a disease that consumes glucose from the CSF. In previous investigations, blood was collected simultaneously with lumbar puncture. The total amount of CSF was about 150 ml (4), the roentgenographic method showed the production of CSF in both lateral ventricles was about 0.6–0.8 ml/min (5–7). So it is widely believed that the CSF is recycled once every 4–6 h, and the CSF glucose level is affected by the serum glucose level 4–6 h before lumbar puncture. Therefore, simultaneous measurement of the blood glucose level is not appropriate to calculate the CSF/blood glucose ratio, especially in patients whose serum glucose level fluctuates greatly over time. In a review of other studies, we found that no studies had examined the CSF/blood glucose ratio in patients with abnormal glucose metabolism or determined how their prior serum glucose level affected their CSF glucose level.

## Materials and methods

We collected clinical data from 195 patients in our department between April 2018 and December 2022. All patients were adults who underwent CSF examination for routine diagnostic and treatment purposes. We collected the patients with demyelinating disease of CNS, autoimmune encephalitis, peripheral neuropathy, primary headache, metabolic encephalopathy, degenerative disease, idiopathic intracranial hypertension, motor neuron disease and the patient whose primary disease had been cured, and patients without neurological diseases (hypertension, electrolyte disturbance, flu). The patients with CSF consumption diseases, such as CNS infection, CNS tumor, prion disease, cerebral infarction, SAH and so on, were excluded. All patients signed an informed consent form to participate in the study.

We determined the CSF glucose and the fingertip blood glucose levels at 6 (6Pre), 5 (5Pre), 4 (4Pre), 3 (3Pre), 2 (2Pre), 1 (1Pre), and 0 h (simultaneous, 0Pre) before lumbar puncture and calculated the average fingertip blood glucose levels at each of these times (Ave). The glucose metabolism state was determined according to the 2018 American Diabetes Association diagnostic criteria (8). We collected patients age, gender, diagnosis and medication data of the patients.

The 195 patients enrolled were divided into two groups: the normal glucose metabolism group (89 patients) and the abnormal glucose metabolism group (106 patients). The abnormal glucose metabolism group included patients with diabetes, impaired fasting blood glucose, and impaired glucose tolerance. Patients with a history of abnormal glucose metabolism and those who completed a glucose tolerance test within 1 year were directly included in groups. Those with no clear glucose metabolism status were required to undergo a 75-g glucose tolerance test.

In the normal glucose metabolism group and the abnormal glucose metabolism group, the patients were divided into three groups depend on their disease: (1) CNS diseases group, (2) disease of peripheral nervous system group, and (3) others group. Others group including patients with motor neuron disease, patient whose primary disease had been cured and patients without neurological diseases.

In the normal glucose metabolism group and the abnormal glucose metabolism group, the patients were divided into two groups according to the use of corticosteroids.

SPSS 22.0 software was used to analyze the data, and a value of *p* ≤ 0.05 was considered significant. The independent-sample T test was used to analyze the effect of age on CSF glucose and the age differences between the normal glucose metabolism group and the abnormal glucose metabolism group. The bivariate correlation analysis method was used to analyze the correlation between CSF glucose and blood glucose levels at each time point and age. Non-parametric test was used to analyze the gender, diagnosis, use of corticosteroids differences between the normal glucose metabolism group and the abnormal glucose metabolism group.

## Results

1. The blood glucose and CSF glucose in the normal glucose metabolism group and the abnormal glucose metabolism group

In the normal glucose metabolism group, the blood glucose level in the 6Pre, 5Pre, 4Pre, 3Pre, 2Pre, 1Pre, and 0Pre groups were 5.51 ± 0.77 mmol/L, 5.57 ± 0.81 mmol/L, 5.54 ± 0.82 mmol/L, 5.86 ± 1.23 mmol/L, 5.99 ± 1.07 mmol/L, 6.50 ± 0.96 mmol/L, 6.41 ± 1.02 mmol/L, respectively. The CSF level was 3.48 ± 0.43 mmol/L.

In the abnormal glucose metabolism group, the blood glucose level in the 6Pre, 5Pre, 4Pre, 3Pre, 2Pre, 1Pre, and 0Pre groups were 7.93 ± 2.66 mmol/L, 7.92 ± 2.51 mmol/L, 7.86 ± 2.58 mmol/L, 7.99 ± 2.61 mmol/L, 8.27 ± 2.67 mmol/L, 8.75 ± 2.95 mmol/L, 8.64 ± 2.83 mmol/L, respectively. The CSF level was 4.39 ± 1.15 mmol/L.

1. Differences of age, gender, diagnosis and medication between the normal glucose metabolism group and the abnormal glucose metabolism group

In the normal glucose metabolism group, the patients average age was 34.37 ± 12.74 years old. In the abnormal glucose metabolism group, the average age was 44.37 ± 14.66 years old. There was no difference in age between the two groups (*F* = 1.688, *p* = 0.195).

There were 49 males in the 89 patients of normal glucose metabolism group, 58 males in the 106 patients of abnormal glucose metabolism group. There was no difference in gender between the two groups (*p* = 0.962).

In the normal glucose metabolism group, there 50 patients in the CNS disease group, 13 patients in the disease of peripheral nervous system group, 26 patients in the others group. In the abnormal glucose metabolism group, there 53 patients in the CNS disease group,17 patients in the disease of peripheral nervous system group, 36 patients in the others group. There was no difference in diagnosis between the two groups (*p* = 0.391).

There were 47 patients use corticosteroids in the normal glucose metabolism group, and 43 patients use corticosteroids in the abnormal glucose metabolism group. There was no difference in the use of corticosteroids between the two groups (*p* = 0.088).

1. Correlation between CSF glucose and age and gender

In the normal glucose metabolism group, we found no relationship between CSF glucose and age (Pearson relationship = −0.096, *p* = 0.373) or gender (*F* = 0.155, *p* = 0.694).

In the abnormal glucose metabolism group, we found no relationship between CSF glucose and age (Pearson relationship = −0.154, *p* = 0.115) or gender (*F* = 0.122, *p* = 0.728).

1. Correlation between CSF and blood glucose levels at each time point before lumbar puncture

In the normal glucose metabolism group, the 95% range of CSF glucose was 2.7–4.3 mmol/L. The bivariate correlation analysis showed that the CSF glucose level increased with the blood glucose level in the 6Pre, 5Pre, 4Pre, 3Pre, 2Pre, 1Pre, and 0Pre groups (*p* < 0.05) (Table 1).

**Table 1:** Correlation analysis between the cerebrospinal fluid glucose level and blood glucose level at each time point before lumbar puncture in patients in the group with normal glucose metabolism.

Blood glucose (mmol/L)	6Pre	5Pre	4Pre	3Pre	2Pre	1Pre	0Pre	AVE
Pearson correlation	0.384	0.419	0.502	0.350	0.287	0.216	0.303	0.561
*P* value	<0.001	<0.001	<0.001	<0.001	0.006	0.042	0.004	<0.001

In the abnormal glucose metabolism group, the 95% range of CSF glucose was 2.7–7.1 mmol/L. The bivariate correlation analysis showed that the CSF glucose level was positively correlated with the glucose levels in the 6Pre, 5Pre, 4Pre, 3Pre, 2Pre, 1Pre, and 0Pre groups (*p* < 0.05) (Table 2).

1. CSF glucose/blood glucose ratio and CSF/average blood glucose ratio at each time point before lumbar puncture

**Figure 1:**
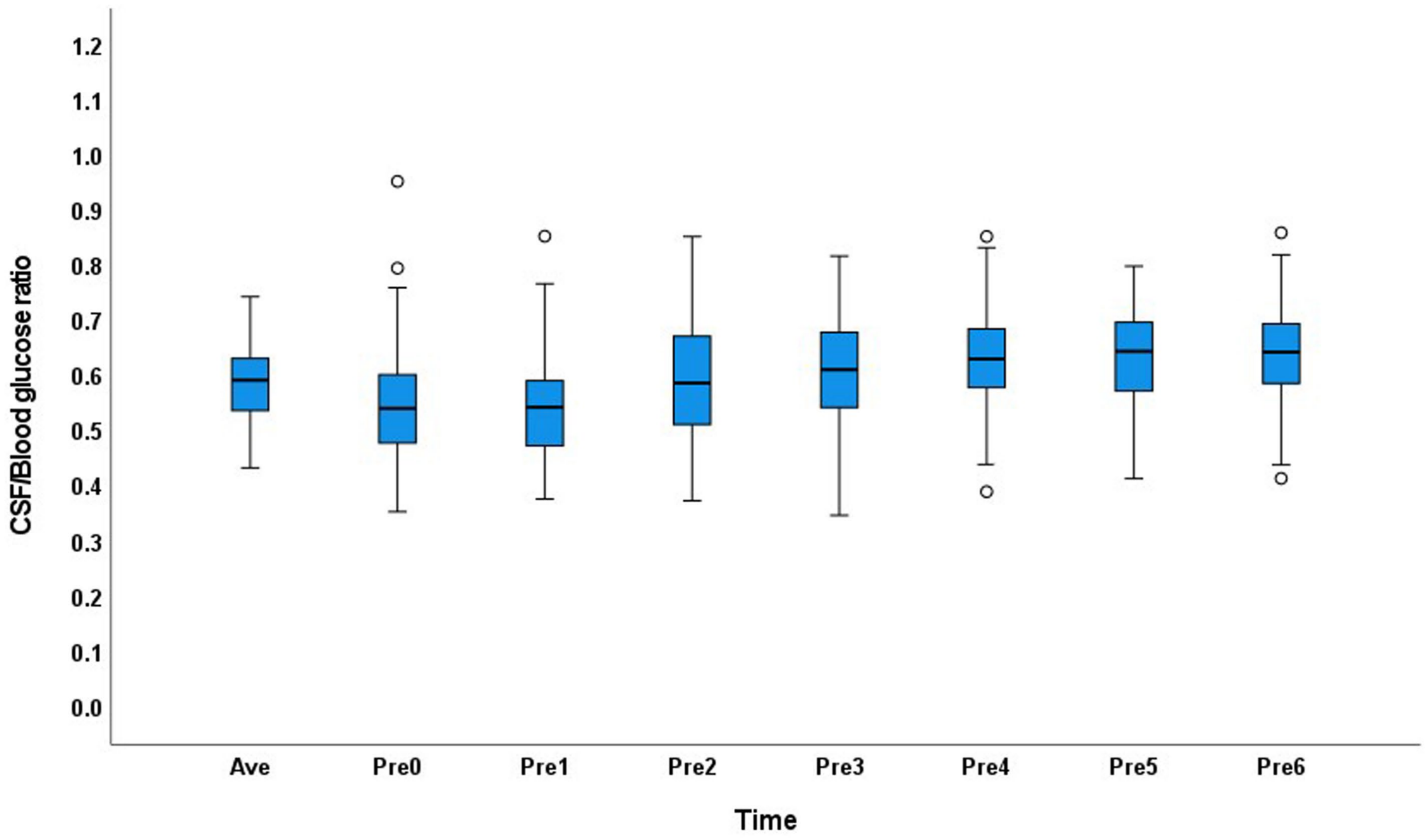
Distribution of the cerebrospinal fluid/blood glucose ratio at each time point and the average value in the normal glucose metabolism group.

In the normal glucose metabolism group, the CSF/blood glucose ratio range was 0.35–0.95 in the 0–6Pre groups, and the CSF/average blood glucose ratio was 0.43–0.74. The distribution of the CSF glucose/blood glucose ratio in each group and the average value showed little variation (Figure 1).

**Figure 2:**
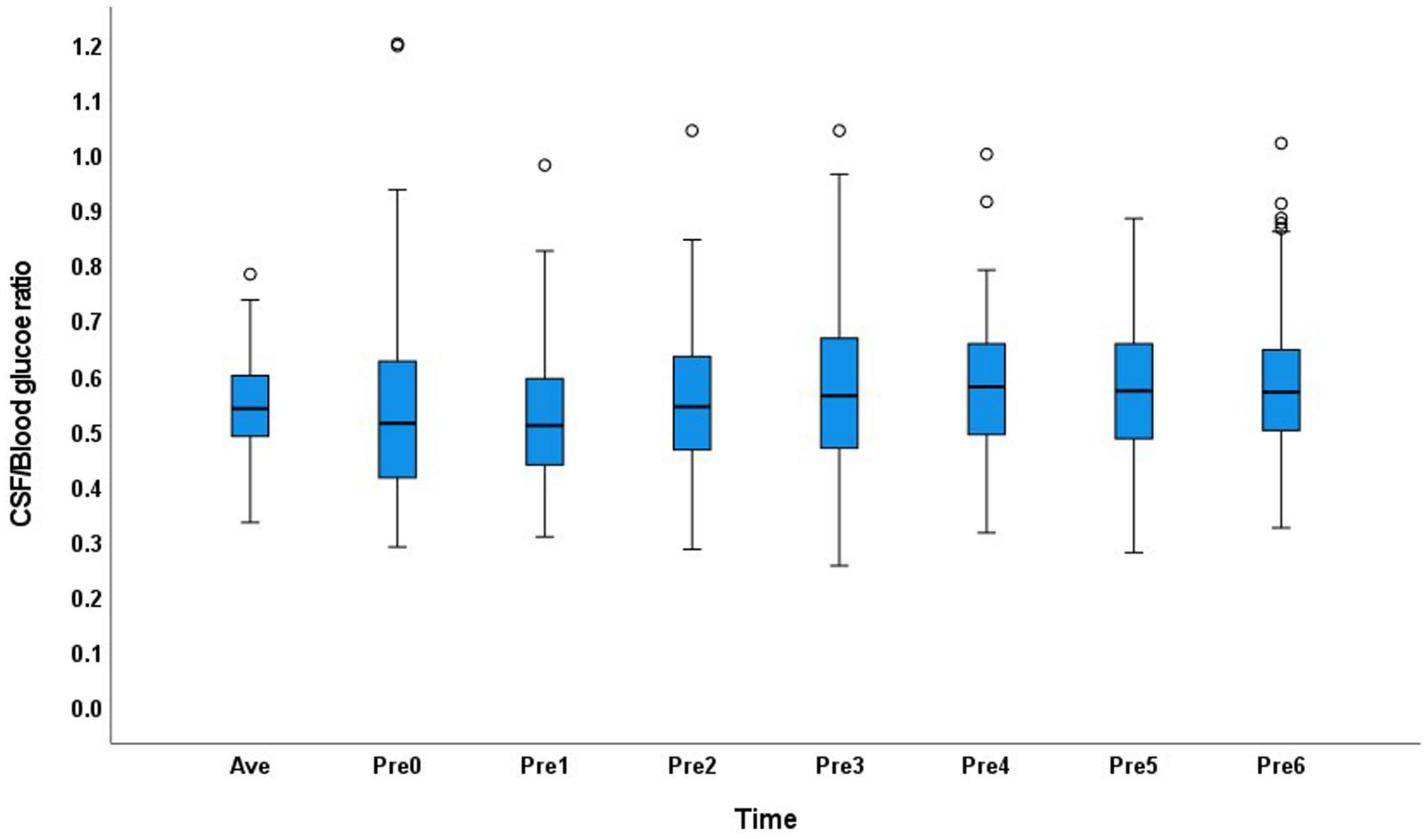
Distribution of the cerebrospinal fluid/blood glucose ratio of each group and the average value in the abnormal glucose metabolism group.

In the abnormal glucose metabolism group, the CSF/blood glucose ratio range was 0.25–1.2 in the 0–6Pre groups, and the CSF/average blood glucose ratio range was 0.33–0.78. The distribution of the CSF/blood glucose ratio across the groups showed greater variation than that in the normal glucose metabolism group (Figure 2).

## Discussion

Glucose is the main energy-providing substance in the CSF. The CSF glucose level declines when energy-consuming diseases are present. Therefore, the CSF glucose level is important in the diagnosis of and evaluation of therapy for infectious CNS diseases, as well as for distinguishing between bacterial and non-bacterial infections (9). Standard reference texts cite the normal CSF glucose level as 2.5–4.4 mmol/L^10^.

The glucose levels of diabetic patients are usually higher than those of people with normal glucose metabolism; therefore, the CSF glucose level should exhibit a similar trend. However, to date, no studies examining normal CSF glucose levels have provided a standard for diabetic patients. Some studies did not consider glucose metabolism (1, 10), while others excluded patients with abnormal blood glucose levels at lumbar puncture (11). The transport of glucose across the blood–brain barrier is mediated by GLUT1 glucose transporter protein (12).Hyperglycemia cannot increase the total GLUT1 glucose transporter, so transport ratio of glucose from blood to CSF decrease with the increase of blood glucose (3, 12, 13). In 2017, a study analyzed the CSF/blood glucose ratio in groups classified according to blood glucose level (14). However, in that study, blood was collected simultaneously with lumbar puncture, which does not account for the delay for glucose in the blood to be transported to the CSF. Moreover, in that study, the blood glucose metabolism classification of the patients was not clear. Therefore, although the study was designed to group subjects according to blood glucose levels, the results were unreliable (Table 2).

**Table 2:** Correlation analysis between the cerebrospinal fluid glucose level and blood glucose level in patients in the group with abnormal glucose metabolism.

Blood glucose (mmol/L)	6Pre	5Pre	4Pre	3Pre	2Pre	1Pre	0Pre	AVE
Pearson correlation	0.632	0.660	0.643	0.613	0.607	0.689	0.528	0.786
*p* value	<0.001	<0.001	<0.001	<0.001	<0.001	<0.001	<0.001	<0.001

Human CSF is circulated every 4–6 h. The CSF collected at a lumbar puncture is produced 4–6 h before the puncture, and the production of glucose in the CSF is related to the blood glucose level. Therefore, in this study, we measured blood glucose levels at different times within 6 h prior to lumbar puncture to analyze the correlation between the CSF glucose and blood glucose levels. The result showed that, in both the normal and abnormal glucose metabolism groups, the CSF glucose level increased with the blood glucose level at 6, 5, 4, 3, 2, 1, and 0 h before lumbar puncture. This study confirmed that the CSF glucose level was influenced by the blood glucose level within 6 h before lumbar puncture, instead of only at the time of puncture. The glucose level in the CSF is closely related to the blood glucose level at 0–6 h before lumbar puncture. This is consistent with previous animal experiments performed by our research group, indicating that the blood glucose level should be monitored in patients 6 h before lumbar puncture (15).

In 2016, a study demonstrated that the blood glucose level increases immediately after food intake, and a period occurred during which the blood glucose level was increased but the CSF glucose level was still low (2). The CSF glucose level fluctuated for approximately 2 h after the blood glucose level changed. This study also demonstrated that the CSF glucose level is influenced by the blood glucose level for a period before lumbar puncture. The CSF glucose level is closely related to the blood glucose level at 2 h before lumbar puncture.

The CSF glucose level depends on the blood glucose level; therefore, it is important to calculate the CSF/blood glucose ratio. The CSF/blood glucose ratio is usually calculated to determine whether the CSF glucose level is normal. The reference range of the CSF/blood glucose level ratio is 0.41–0.88, with an average of 0.6 (1, 10, 11, 14). In this study, we calculated the CSF/blood glucose ratio at each time point and the average value.

Wilhelmina and his colleagues studied 8,871 CSF samples, they found after the age of 6 months, CSF glucose gradually increased over the entire age (11). In our study, the CSF glucose was not correlated with age. There were two possible reasons for the inconsistent results: (1) maybe the previous study did not take glucose metabolism into account; and (2) due to the amount of data in our study is relatively small. CSF glucose was not influenced by gender in our study, which was consistent with previous study (16). In our study, there were no differences in age, gender, diagnosis and the use of corticosteroids.

In clinical practice, not every patient could be fast or prohibit the use of anti-diabetic drug for 6 h before lumbar puncture. We did not prohibit the patients from eating food or taking medications. Maybe the patient just had dinner, maybe it is time for anti-diabetic drug, maybe the patient is fasting, maybe the patient is taking parenteral nutrition, in any situation, we hope we can get accurate CSF/blood glucose ratio as soon as possible.

In the normal glucose metabolism group, the CSF/average blood glucose ratio range was similar in the 0–6Pre groups, and the CSF/blood glucose ratio in different groups showed little variation. This may have occurred because the blood glucose level in people with normal glucose metabolism is relatively stable. In this study, the 95% range of the CSF glucose level in patients with normal glucose metabolism was 2.7–4.3 mmol/L, and the normal range is 2.5–4.4 mmol/L^10^. Therefore, in people with normal glucose metabolism, the absolute value of the CSF glucose level can be compared with the normal range to determine whether the CSF glucose level is normal. If the CSF glucose level is <2.7 mmol/L in an individual with normal glucose metabolism, CNS infection or carcinoma should be considered.

Blood glucose levels fluctuate greatly after food intake in patients with abnormal glucose metabolism. If blood is obtained when patients have a high blood glucose level, a false low CSF/blood glucose ratio will be obtained, and the patient may be misdiagnosed with CNS infection or tumor. However, anti-diabetic medication can also lead to a decrease in the blood glucose level over a short time. If the CSF glucose level is compared with a low blood glucose level, a false high or normal CSF/blood glucose ratio can be obtained even in patients with diseases that consume CSF glucose; therefore, a diagnosis of CNS infection or tumor may be missed. However, in a literature search, we found no articles examining the CSF/blood glucose ratio in patients with abnormal glucose metabolism.

In patients with abnormal glucose metabolism, the 95% range of CSF glucose was 2.7–7.1 mmol/L, which is much higher than the normal range of 2.5–4.4 mmol/L^10^. False negative results may be obtained in patients with CNS infection. Therefore, the blood glucose level at a single point cannot be used as a reference to determine whether the patient’s CSF glucose level is decreased. The CSF/blood glucose ratio distribution showed discrete values at each time point. Therefore, the average blood glucose level should be obtained 6 h before lumbar puncture to calculate the CSF/blood glucose ratio in patients with abnormal glucose metabolism. Since the blood glucose fluctuate greatly, we would better measure blood glucose as much as possible before lumbar puncture to calculate the CSF/average blood glucose ratio in the abnormal glucose metabolism patients. Considering the clinical feasibility, fingertip blood should be collected at 6, 5, 4, 3, 2, 1, and 0 h before lumbar puncture to determine the average blood glucose level to compare with the CSF glucose level. The development of the non-invasive blood glucose detection technology may be helpful in this situation.

Diabetes is one of the top 10 leading causes of death worldwide. Additionally, patients with abnormal glucose metabolism are more susceptible to infectious diseases. Therefore, glucose metabolism screening should be emphasized, especially when an infectious disease of the CNS is suspected. If the glucose metabolism status of a patient is unclear or abnormal, fingertip blood should be collected at 6, 5, 4, 3, 2, 1, and 0 h before lumbar puncture, and the CSF/average blood glucose ratio can be used to assist in the diagnosis. Further more, the CSF lactate level is also an important indicator of the CSF metabolism (17). We can combine the CSF glucose metabolism and lactate level to determine the CSF energy metabolism to improve diagnostic accuracy.

## Conclusion

The CSF glucose level is influenced by the blood glucose level. Human CSF is circulated every 4–6 h. Therefore, the CSF glucose level determined at a single time point depends on the blood glucose level at 6 h before lumbar puncture. In patients with normal glucose metabolism, the blood glucose level exhibits a relatively stable range. Therefore, the absolute value of the CSF glucose can be used to determine whether the glucose level is normal. For patients with abnormal glucose metabolism, the blood glucose level fluctuates, and no single time point can accurately represent the blood glucose level. Therefore, the average blood glucose level should be obtained at each hour before lumbar puncture to calculate the CSF/blood glucose ratio. Fingertip blood can be obtained at 6, 5, 4, 3, 2, 1, and 0 h before lumbar puncture to determine the average blood glucose level. If the patient’s glucose metabolism state is unclear, a glucose tolerance test should be performed or the average blood glucose level should be calculated according to the method used for patients with abnormal glucose tolerance.

### Statements

The work is original and has not been previously published, whole or in part, in any language. The material is not currently, and will not be, under simultaneous consideration for publication elsewhere while under consideration at this journal.

## Data availability statement

The raw data supporting the conclusions of this article will be made available by the authors, without undue reservation.

## Ethics statement

The studies involving human participants were reviewed and approved by the Ethical Committee of the Chinese PLA General Hospital. The patients/participants provided their written informed consent to participate in this study. Written informed consent was obtained from the individual(s) for the publication of any potentially identifiable images or data included in this article.

## Author contributions

QT and XX collected the data, performed the statistical analysis, and wrote the manuscript. JZ took full responsibility for the whole study and revised the manuscript. MH, YM, LW, XW, HW, and SY helped collect and interpret the data. All authors contributed to the article and approved the submitted version.

## Conflict of interest

The authors declare that the research was conducted in the absence of any commercial or financial relationships that could be construed as a potential conflict of interest.

## Publisher’s note

All claims expressed in this article are solely those of the authors and do not necessarily represent those of their affiliated organizations, or those of the publisher, the editors and the reviewers. Any product that may be evaluated in this article, or claim that may be made by its manufacturer, is not guaranteed or endorsed by the publisher.
