# Corrigendum: Correlation between blood glucose and cerebrospinal fluid glucose levels in patients with differences in glucose metabolism

**DOI:** 10.3389/fneur.2023.1239968

**Published:** 2023-07-03

**Authors:** Qing Che Tan, Xiao Wei Xing, Jia Tang Zhang, Mian Wang He, Yu Bao Ma, Lei Wu, Xiaolin Wang, Hong Fen Wang, Sheng Yuan Yu

**Affiliations:** ^1^Medical School of Chinese PLA, Beijing, China; ^2^First Affiliated Hospital of Chinese PLA General Hospital, Beijing, China; ^3^Hainan Branch of People's Liberation Army General Hospital, Sanya, China

**Keywords:** correlation, cerebrospinal fluid glucose, blood glucose, central nervous system infection, CSF/blood glucose ratio, abnormal glucose metabolism, normal glucose metabolism

In the published article, there was an error in the affiliations as published. The affiliation for the first author “Qing Che Tan” was published as “1. Medical School of Chinese PLA, Beijing, China” but should be “1. Medical School of Chinese PLA, Beijing, China, 2. First Affiliated Hospital of Chinese PLA General Hospital, Beijing, China”.

The authors apologize for this error and state that this does not change the scientific conclusions of the article in any way. The original article has been updated.

